# Supplementary material for: Single-Cell Network-Based Drug Repositioning for Discovery of Therapies against Anti-Tumour Necrosis Factor-Resistant Crohn’s Disease
Source: Int J Mol Sci. 2023 Sep 14;24(18):14099. doi: 10.3390/ijms241814099 (PMC10531326; doi:10.3390/ijms241814099)

Supplementary figure S1. Differences of cellular heterogeneity between the inflamed colonic tissue (Involved) normal colonic tissue (Uninvolved) during anti-TNF therapy in CD.

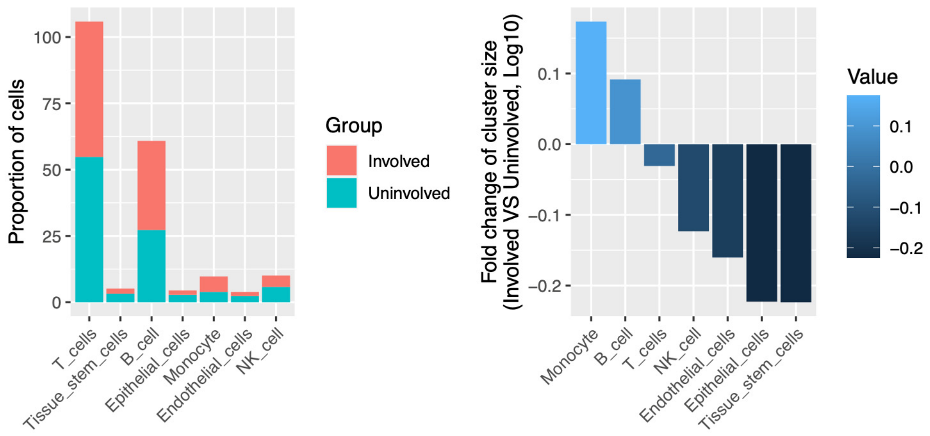

Supplementary figure S2. The drug scores for the anti-TNF resistance in all samples among top-ranked significant drugs, according to both edgeR and limma algorithms (FDR < 0.05).

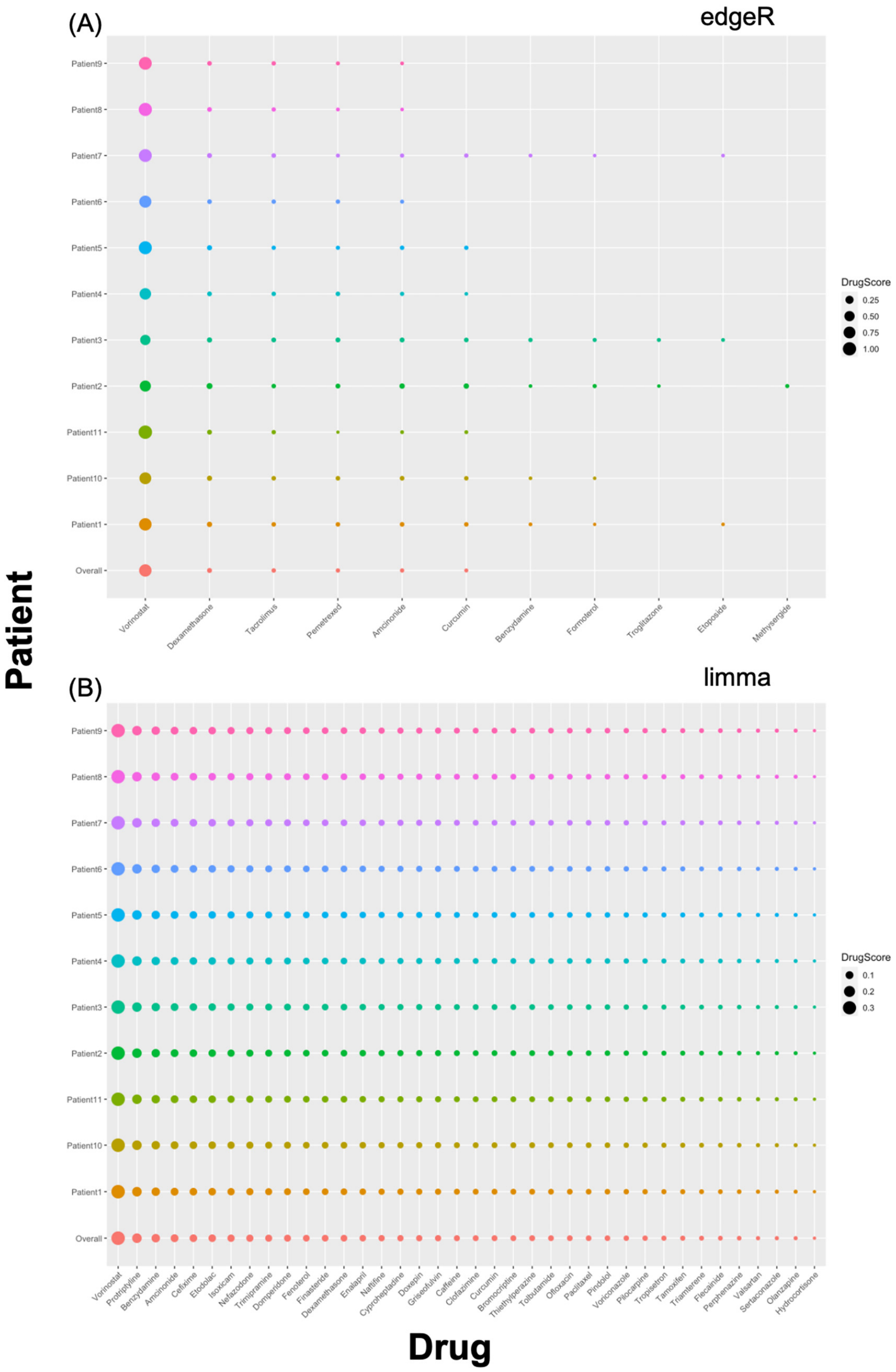

Supplement: Supplementary file 1 [file ijms-24-14099-s001.zip › ijms-2602612-supplementary.pdf]
